# Biomarkers in Acute Myocarditis and Chronic Inflammatory Cardiomyopathy: An Updated Review of the Literature

**DOI:** 10.3390/jcm12237214

**Published:** 2023-11-21

**Authors:** Giulia Crisci, Emanuele Bobbio, Piero Gentile, Daniel I. Bromage, Entela Bollano, Emma Ferone, Muhammad Zubair Israr, Liam M. Heaney, Christian L. Polte, Antonio Cannatà, Andrea Salzano

**Affiliations:** 1 Department of Translational Medical Sciences, Federico II University, 80131 Naples, Italy; criscigiulia21@gmail.com; 2 Italian Clinical Outcome Research and Reporting Program (I-CORRP), 80131 Naples, Italy; 3 Department of Cardiology, Sahlgrenska University Hospital, 41345 Gothenburg, Sweden; emanuele.bobbio@vgregion.se (E.B.); entela.bollano@vgregion.se (E.B.); 4 Institute of Medicine, The Sahlgrenska Academy at the University of Gothenburg, 41390 Gothenburg, Sweden; christian.polte@vgregion.se; 5 De Gasperis Cardio Center, Niguarda Hospital, 20162 Milan, Italy; pierogentile.87@gmail.com; 6 Department of Cardiology, King’s College Hospital NHS Foundation Trust, Denmark Hill, London SE5 9RS, UK; daniel.bromage@kcl.ac.uk (D.I.B.); emma.ferone@kcl.ac.uk (E.F.); 7 Department of Cardiovascular Sciences, Faculty of Life Sciences & Medicine, King’s College London, London SE5 8AF, UK; 8 Department of Cardiovascular Sciences, University of Leicester and NIHR Leicester Biomedical Research Centre, Groby Road, Leicester LE3 9QP, UK; mzi3@leicester.ac.uk; 9 School of Sport, Exercise and Health Sciences, Loughborough University, Loughborough LE11 3TU, UK; l.m.heaney2@lboro.ac.uk; 10 Department of Clinical Physiology, Sahlgrenska University Hospital, 41345 Gothenburg, Sweden; 11 Cardiology Unit, AORN A Cardarelli, 80131 Naples, Italy

**Keywords:** myocarditis, biomarkers, heart failure, inflammatory heart disease, cardiac sarcoidosis, imaging

## Abstract

Myocarditis is a disease caused by cardiac inflammation that can progress to dilated cardiomyopathy, heart failure, and eventually death. Several etiologies, including autoimmune, drug-induced, and infectious, lead to inflammation, which causes damage to the myocardium, followed by remodeling and fibrosis. Although there has been an increasing understanding of pathophysiology, early and accurate diagnosis, and effective treatment remain challenging due to the high heterogeneity. As a result, many patients have poor prognosis, with those surviving at risk of long-term sequelae. Current diagnostic methods, including imaging and endomyocardial biopsy, are, at times, expensive, invasive, and not always performed early enough to affect disease progression. Therefore, the identification of accurate, cost-effective, and prognostically informative biomarkers is critical for screening and treatment. The review then focuses on the biomarkers currently associated with these conditions, which have been extensively studied via blood tests and imaging techniques. The information within this review was retrieved through extensive literature research conducted on major publicly accessible databases and has been collated and revised by an international panel of experts. The biomarkers discussed in the article have shown great promise in clinical research studies and provide clinicians with essential tools for early diagnosis and improved outcomes.

## 1. Introduction

**Figure 1:**
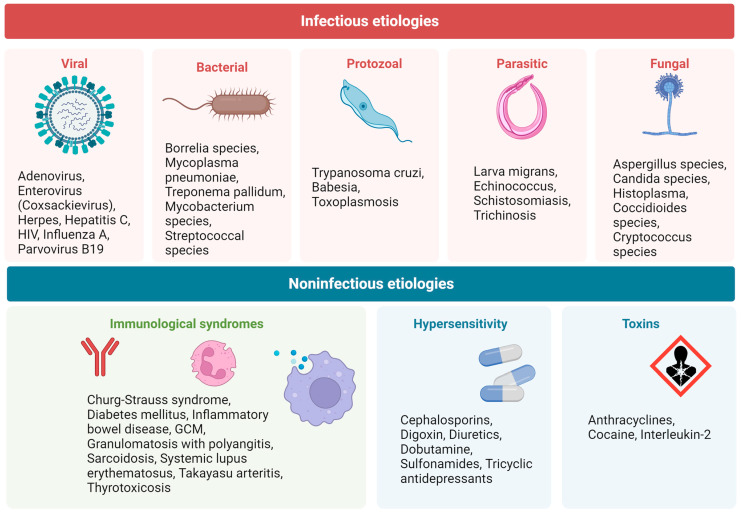
Different etiologies implicated in the development of myocarditis. Abbreviations: GCM, Giant cell myocarditis; HIV, Human immunodeficiency virus.

Myocarditis is a heterogeneous inflammatory disease of the myocardium caused by a wide range of factors, including infectious triggers as well as autoimmune disorders, hypersensitivity reactions, and toxins [1,2,3]. However, viral infections have been documented to constitute the most prevalent cause of myocarditis, particularly in children. (Figure 1) [2,3]. Inflammatory cardiomyopathy (ICM) is defined as myocarditis in association with ventricular remodeling and cardiac dysfunction [4]. As most cases of myocarditis resolve spontaneously—with about 70% of patients recovering myocardial function in the first year—the incidence and prevalence of this condition are difficult to estimate, and the diagnosis is seldom verified with an endomyocardial biopsy (EMB) [5]. Nevertheless, myocarditis and ICMs are noteworthy entities, and fulminant forms are still a common cause of cardiogenic shock in young people [6,7].

**Figure 2:**
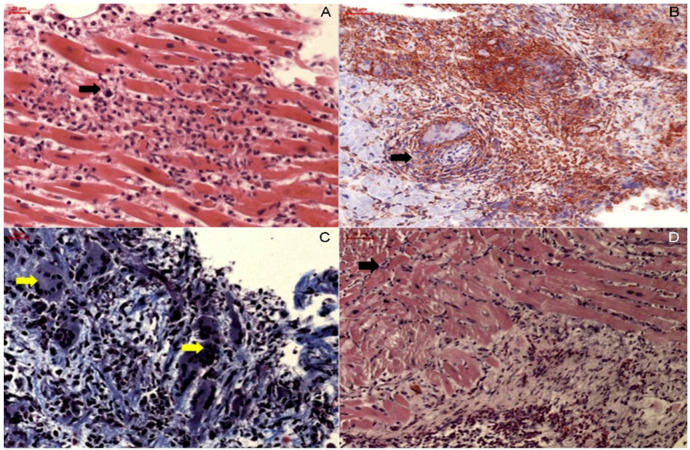
Different types of acute myocarditis. (**A**) Acute lymphocytic myocarditis with focal inflammatory cell infiltrates (black arrow) and cardiomyocyte necrosis. (**B**) Cardiac sarcoidosis, with evidence of granuloma (black arrow). (**C**) Giant cell myocarditis, with presence of giant multinucleated cells (yellow arrows). (**D**) Eosinophilic myocarditis. Re-used with permission from Dominguez et al. [8].

Myocarditis can be characterized based on etiology, phase, disease severity, pathological findings, and predominant symptoms. Acute myocarditis is characterized by a short amount of time elapsed from the onset of symptoms to diagnosis (within one month), while chronic ICM is a progression of acute myocarditis associated with established dilated cardiomyopathy or other types of established myocardial dysfunction and a longer duration of symptoms [3,5]. Myocarditis can also be classified as eosinophilic, lymphocytic, giant cell, or granulomatous, according to the dominating cell type infiltrating the myocardium (Figure 2) [4].

The underlying mechanisms that lead to myocarditis and its progression from acute inflammation to chronic dysfunction are still not fully understood. Some viruses, such as Coxsackie B viruses, have been shown to cause direct tissue damage in animal models, while others, such as parvovirus B19, have not been studied in this context [9,10,11]. There is also some evidence to suggest that genetic abnormalities may play a role in susceptibility to myocarditis, but further research is needed to understand this association [12,13].

No comprehensive population-based epidemiological study has been able to thoroughly record the wide range of clinical presentations seen in both acute and chronic myocarditis. This is primarily due to two key factors: the constantly changing and varied symptoms that patients exhibit, which continue to pose challenges for healthcare professionals attempting to make differential diagnoses, and the absence of reliable non-invasive tests, which hampers the ability to establish definitive diagnoses or provide precise prognostic information. In clinical practice, physicians rely on a combination of symptoms, laboratory findings, and imaging characteristics to diagnose myocarditis. The gold standard for diagnosing suspected myocarditis remains EMB [4,14]. However, questions remain regarding the necessity of performing routine biopsies in case of suspected myocarditis, particularly given the significant advancements in imaging technology. Classically, for the WHO/IFC diagnostics criteria for myocarditis, a proven histological involvement of the myocardium was mandatory [4]. However, cardiac magnetic resonance imaging (CMRI) today represents a powerful and not invasive tool, allowing the identification of myocardial inflammation and emerging as an essential instrument in myocarditis diagnosis [3]. For this reason, it has been suggested that EMB should be limited only to high-risk patients (based on clinical data such as blood pressure, symptoms of HF, echocardiographic and ECG findings), suggesting the use of CMRI in all other cases (i.e., low and intermediate risk patients) [3].

Multiple biomarkers have been extensively studied, with increasing evidence accumulating to support their clinical usefulness in acute myocarditis and ICM [15]. Biomarkers are routinely used in clinical practice in several cardiovascular disorders, and a continuum has been defined, with a role (possible or definite) for community-based screening, diagnosis, risk stratification/prognosis, phenotyping, management/monitoring, and treatment [16]. However, none of the novel biomarkers has demonstrated sufficient impact alone in the management of acute myocarditis and ICM. Thus, it has been suggested that the integration of multiple biomarkers may represent a promising novel strategy, particularly when biomarkers from diverse pathophysiological patterns or different techniques are combined (e.g., a combination of circulating and imaging biomarkers) [17,18].

In 2001, the Biomarkers and Surrogate End Point Working Group aimed to standardize the (mis)use of the term ‘biomarker’, suggesting the following definition be used: “a characteristic that is objectively measured and evaluated as an indicator of normal biological processes, pathogenic processes, or pharmacologic responses to a therapeutic intervention” [19]. Therefore, in current practice, the term ‘biomarker’, which includes emerging tools, technologies, and strategies intended to improve the knowledge about a disease, can be applied to all markers derived from blood, urine, genetic samples, imaging, physiological tests, and tissue-specimen biopsies [20].

The purpose of the present article is to review the biomarkers currently known to associate with these conditions and to evaluate those that have shown great promise in clinical research studies. This is intended to provide a simple and accessible source of information for clinicians to use around their demanding daily schedules.

## 2. Acute Myocarditis and Chronic Inflammatory Cardiomyopathy: A General Overview

### 2.1. Definition, Diagnostic Approach, and Treatment

Myocarditis presents heterogeneous signs and symptoms, ranging from subclinical disease to ventricular fibrillation, refractory cardiogenic shock, or sudden cardiac death [1]. Chest pain, dyspnea, and fatigue are the most common symptoms in patients with myocarditis [1,21,22]. Fever is also common, seen in about 65% of patients, whereas other prodromal manifestations such as flu-like symptoms, stomach problems, sore throat, or respiratory infections may occur a few days or weeks before the acute phase and can affect 18% to 80% of patients [3,21].

The treatment for myocarditis and ICM is primarily supportive and follows the standard medical therapy for other forms of dilated cardiomyopathies [3]. Patients with systolic dysfunction should receive guideline-directed medical and device treatment for heart failure (HF) [23]. In severe cases of HF, inotropic support or short-term mechanical circulatory assistance may be necessary [24,25]. The use of immunosuppression in myocarditis is still a controversial topic and is recommended only in biopsy-proven autoimmune myocarditis and ICM refractory to standard therapy [3,5]. People with acute myocarditis and mild heart infections usually recover without any lasting effects, but it is recommended that they avoid heavy physical activity or sports for at least 3 months due to an increased risk of ventricular arrhythmias [4].

### 2.2. Biomarkers

Recommended laboratory tests to identify patients with myocarditis include a complete blood count, myocardial necrosis biomarkers (high-sensitivity cardiac troponin assays, and creatine kinase-MB (CK-MB)), and nonspecific inflammatory biomarkers (C reactive protein, erythrocyte sedimentation rate) [4]. Peripheral blood serological and virological tests are rarely informative, except in the case that a certain etiology is suspected (albeit not specific) [5,15].

Electrocardiogram (ECG) tests are abnormal in around 85% of patients, with the most common abnormality being ST-segment elevation, mimicking acute coronary syndromes [22]. High-degree atrioventricular blocks (AVB), symptomatic bradycardia, or ventricular arrhythmias would increase the suspicion of more aggressive forms [3,24].

Echocardiography is still considered the first-line diagnostic tool in patients with clinically suspected myocarditis [4]. Although echocardiographic findings are often nonspecific in these patients, echocardiography aids in ruling out other differential diagnoses and is most likely useful for longitudinal follow-up studies [6].

**Figure 3:**
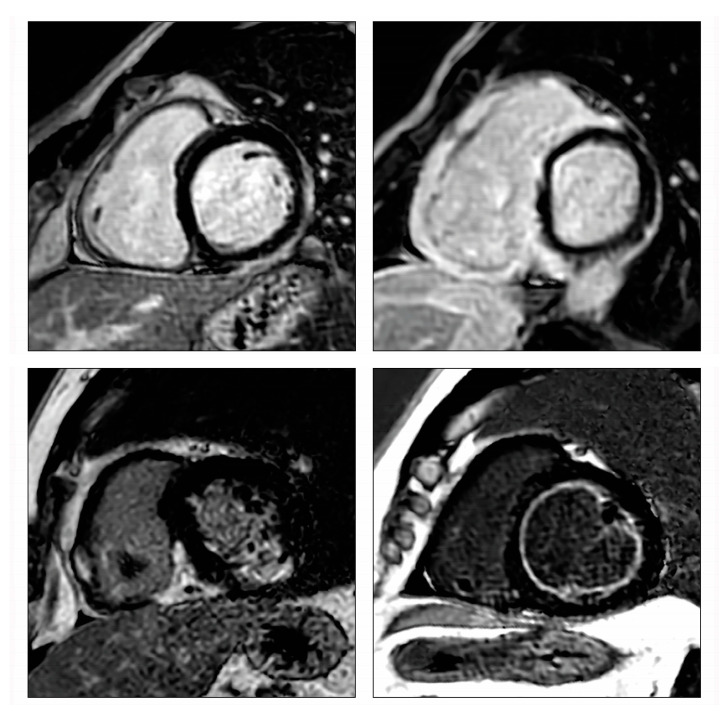
Characteristic late gadolinium enhancement (LGE) patterns in myocarditis. Characteristic late gadolinium enhancement (LGE) patterns in viral myocarditis (**upper left**) inferolateral subepicardial LGE), giant cell myocarditis (**upper right**) complex LGE involving both ventricles including the right ventricular insertion points), cardiac sarcoidosis (**lower left**) complex LGE involving both ventricles including the inferior right ventricular insertion point), and eosinophilic myocarditis (**lower right**) diffuse subendocardial LGE with high signal intensity). Re-used with permission from Polte et al. [27].

Cardiac magnetic resonance imaging (CMRI) has emerged as a powerful non-invasive diagnostic tool, mainly due to its multiparametric tissue characterization ability, and has been validated using EMB as a reference (Figure 3) [26,27,28]. CMRI is recommended in patients with clinically suspected myocarditis or in patients with chest pain, elevated troponin levels, and normal coronary arteries [26]. The ‘Updated Lake Louise Criteria’, a consensus guide enabling the CMRI-based diagnosis of myocarditis, focuses on the visualization of several hallmarks associated with myocardial inflammation: edema, hyperemia, capillary leak, myocardial necrosis, and fibrosis [26,29]. A high likelihood of myocarditis is assumed if “2 out of 2” diagnostic criteria are fulfilled: one positive T2-based criterion (T2-weighted imaging or T2 mapping) and one T1-based criterion (T1-mapping, extracellular volume, or late gadolinium enhancement (LGE)) [26,29]. CMRI conveys not only diagnostic but also prognostic information in patients with myocarditis [30,31].

Fluorine-18 fluorodeoxyglucose (^18^F-FDG) positron emission tomography/computed tomography (PET/CT) can visualize myocardial inflammation and has become an established diagnostic tool in the complicated workup of patients with clinically suspected cardiac sarcoidosis [32,33,34,35]. Furthermore, the method might also be useful in other complicated cases with an inconclusive CMRI and/or EMB study, for instance, in recurrent myocarditis [27].

EMB is widely regarded as the reference standard in diagnosing myocarditis, but it is also an invasive procedure that is associated with an increased risk of complications [36]. In high-volume centers, cardiac complications have been described in 1–2% of patients, but this figure can be as high as 9% at centers with less experience [37,38]. The accuracy of EMB is somewhat limited, as the sample sites may not always align with the spread of inflammation [39].

## 3. Specific Forms of Myocarditis

### 3.1. Infectious Myocarditis

#### 3.1.1. Definition, Diagnostic Approach, and Treatment

Viral infections are presumed to be the most common cause of myocarditis in Europe and North America [2,5]. The most commonly encountered viruses are enterovirus (Coxsackie B virus), parvovirus, Epstein–Barr virus (EBV), and adenovirus [2,5]. Other aetiological agents that can cause myocarditis include hepatitis C virus, influenza virus, and human immunodeficiency virus (HIV), as well as several bacteria and protozoans, including Trypanosoma cruzi (Chagas’ disease) [9]. In rare cases, the spirochete bacterium Borrelia burgdorferi can cause myocarditis, which mainly affects the conduction system of the heart and results in AVB [2,5].

**Figure 4:**
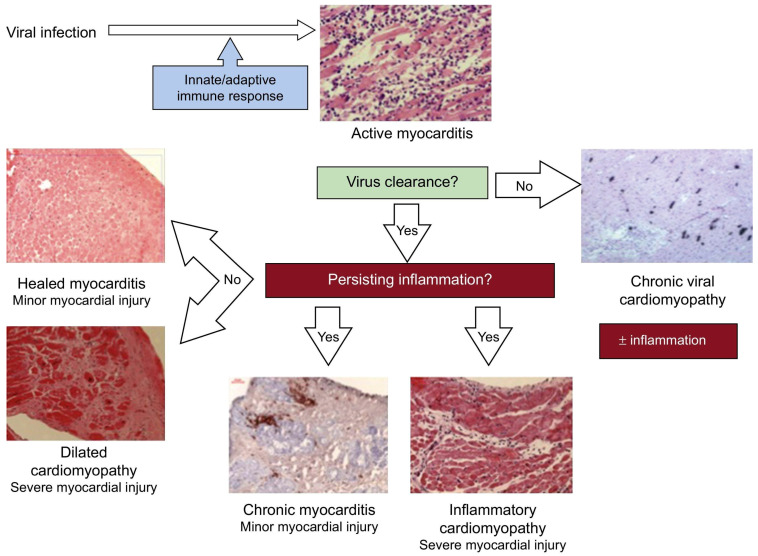
Pathogenesis of viral and inflammatory cardiomyopathy. Re-used with permission from Dominguez et al. [8].

Virus-induced acute myocarditis can refer to both virus-mediated myocarditis and virus-triggered myocarditis. Respiratory viruses, for example, are common viruses that could trigger immune-mediated lymphocytic myocarditis in the absence of a viral genome in the myocardium [40]. In predisposed individuals, molecular mimicry between cardiac and viral antigens, which can result in autoreactive T-cell infiltration in the myocardium, is suspected to be the underlying mechanism of myocardial injury in virus-triggered myocarditis (Figure 4). The severity and course of the disease, as well as treatment options, are dependent on the underlying cause. If a viral origin is suspected, various tests, such as viral culture from peripheral specimens like urine or stool, viral antibody levels, and serological tests, have been utilized to identify the specific pathogen [6,15]. However, these methods have limitations. Some viruses (i.e., Parvovirus B19) may be the cause of both systemic infections associated with myocarditis and chronic ICM [41]. The viral genome can be detected both in plasma and myocardium in myocarditis, while it is generally detected only in the myocardium of chronic ICM, even if with different titers [41,42]. Still, low copy numbers of the viral genome may reflect latent infection and should be interpreted as a bystander since they can also be found in normal myocardium [43]. The resolution of the viral syndrome with the extinction of viral antigens could explain the frequent self-resolving natural history of most acute myocarditis.

Chagas’ disease, also known as American trypanosomiasis, is prevalent in many countries within Central and South America [6]. This disease is caused by the parasite Trypanosoma cruzi, which is transmitted to humans by various species of triatomine bugs. Trypanosoma cruzi can cause both acute myocarditis and chronic inflammation, leading to severe cardiomyopathy and advanced HF [6]. Currently, there is no cure for the disease, and treatment is mainly supportive.

Rheumatic heart disease, a systemic immune process provoked by a beta-hemolytic streptococcal infection, represents the most frequently acquired heart disease in developing countries [44]; despite it classically involving valves, and it has been reported that at least 10% of patients develop a secondary HF due to valve involvement, recent evidence showed that 25–30% of subjects undergoing heart transplantation had non-diagnosed myocarditis, leading to refractory heart failure and, ultimately, heart transplantation [45].

#### 3.1.2. Biomarkers

##### Circulating Biomarkers

In clinical practice, measuring levels of biomarkers in patients presenting with suspected myocarditis may still aid in confirming a diagnosis of myocarditis. The use of blood cell count, erythrocyte sedimentation rate, and C-reactive protein is widespread and easy to obtain; however, these are nonspecific inflammatory biomarkers, and their increase may be attributed to any inflammatory condition.

Traditional biomarkers of myocardial cell injury classically increase in infective myocarditis. However, their increase is nonspecific, with many patients displaying normal levels [46]. Cardiac troponin T (cTnT) and I (cTnI)—both classic and high sensitivity (hs) assays—are among the biomarkers investigated and have demonstrated improved sensitivity in detecting cardiac damage in infective myocarditis compared to other markers such as CK and CK-MB [46]. Similarly, natriuretic peptides (NPs), reflecting the response to the stretching of myocardial cells, are frequently elevated in infective myocarditis, but their levels may also be normal at the time of clinical presentation, limiting their usefulness in diagnosis [46]. Nonetheless, increased levels of NPs have been associated with poor prognosis [46].

In clinical practice, the measurement of anti-viral antibody levels is often used despite being discouraged by current guidelines due to its lack of diagnostic utility. Indeed, levels of IgM and IgG can increase nonspecifically in viral infections, even in the absence of cardiac involvement. Furthermore, serum levels of antibodies directed against the cardiotropic virus are found at high levels worldwide [47].

Direct virus isolation is of marginal value in confirming the diagnosis. Virus serology, indeed, does not consent to the diagnosis of myocarditis, neither in patients with suspect myocarditis nor in patients with confirmed histological signs of myocardial inflammation. Thus, EBM remains the gold standard technique [47].

More information is available regarding chronic Chagas cardiomyopathy. Elevated levels of CA-125, uric acid, and C-reactive protein were found to be associated with poor outcomes [48]. Similarly, increased levels of interleukin (IL)-8, IL-1b, and IL-12 were shown to be associated with a worse prognosis in HF [49]. In addition, low levels of micro-RNA (miRNA) 223-5p, possibly through the pathways related to receptor tyrosine kinases, have recently been associated with the severity of Chagas cardiomyopathy and worse left ventricular (LV) ejection fraction (EF) [50].

##### Imaging Biomarkers

Echocardiography plays a crucial role in the evaluation and risk stratification of patients with suspected infective myocarditis. The estimation of the LVEF and of subregional contractility, with EF impairment and local hypokinesia, especially in inferior and inferior-lateral walls, are associated with a worse prognosis [21,22]. In addition, myocardial damage may lead to diastolic dysfunction [21,22].

CMRI is a diagnostic tool widely used and allows the recognition of the hallmarks of myocardial inflammation following the “Updated Lake Louise Criteria”, as previously described [26,29]. Classically, the LGE pattern involves the subepicardial and/or mid-wall layers of the myocardium, predominately in the basal to mid-lateral and inferolateral wall segments of the left ventricle [27].

Recently, it has been shown that about 70% of patients with rheumatic heart disease display a positive 18F-FDG PET/CT and that 90% of patients have a positive uptake of t-gallium-67 cardiac scintigraphy, regardless of LVEF [44].

### 3.2. COVID-19 and Post-Vaccination Associated Myocarditis

#### 3.2.1. Definition, Diagnostic Approach, and Treatment

The Coronavirus disease 2019 (COVID-19) is caused by the severe acute respiratory syndrome coronavirus 2 (SARS-CoV-2) and can result in a range of cardiac manifestations, including myocardial injury [51,52]. Several cases of suspected myocarditis have been reported, but only a few have been confirmed through histological evidence of lymphocytic infiltration or detection of the SARS-CoV-2 genome [52]. Currently, the evidence suggests that viral myocarditis is a rare complication in patients with COVID-19 (4); the exact mechanism of how it develops is still debated [52]. Myocardial inflammation can involve just a local cardiac area, or a wide area of myocardium, leading to severe decompensated HF or cardiogenic shock (5). It has been demonstrated that about one-third of acute HF in patients affected by COVID-19 is due to myocarditis or stress cardiomyopathy (3).

Myocarditis after receiving the mRNA COVID-19 vaccine is a rare side effect, predominantly described in male adolescents and young adults [53,54]. Vaccine-induced myocarditis has also been considered a form of immune reaction to components of vaccines; more frequent in men, with ages ranging from 17 to 52 years, it is usually of a benign nature [54].

#### 3.2.2. Biomarkers

None of the known biomarkers has been specifically described to have a role in SARS-CoV-2-related myocarditis nor in post-vaccination myocarditis. Despite being described as elevated, troponins and natriuretic peptides can also be normal. Thus, even if not elevated, myocarditis cannot be excluded [55]. Despite the presence of inflammation, some cases have been described where C-reactive protein is at normal levels [56]. The same is seen for ECG alterations, which display high variability (from alterations of ST segments, atrioventricular conduction impairment, QT prolongations, and no evidence of abnormality) [57].

CMRI is the mainstay in the diagnostic workup and shows signs of myocardial inflammation, which are often classic in cases of viral myocarditis, especially when following mRNA COVID-19 vaccination. Myocarditis following mRNA COVID-19 vaccination could also be visualized using somatostatin receptor PET/CT, a promising method for molecular inflammation imaging [27,58].

### 3.3. Sarcoidotic Myocarditis

#### 3.3.1. Definition, Diagnostic Approach, and Treatment

Sarcoidosis is a systemic inflammatory disease of unknown etiology characterized by non-caseating granuloma formation [59]. Accumulating evidence suggests that sarcoidosis is caused by an immune-mediated response to an unidentified antigenic trigger in genetically susceptible individuals [2,59]. Most cases occur in patients 25 to 60 years of age, particularly in women [59,60]. Organ involvement is variable. Clinical heart disease has been confirmed in around 5% of patients with systemic sarcoidosis, but autopsy studies found that clinically manifested disease may represent just the tip of the iceberg [2,5]. Symptoms of sarcoidotic myocarditis are heterogeneous and may range from silent myocardial granulomas to progressive HF, symptomatic conduction disturbances, and ventricular arrhythmias [61]. This can make the diagnosis difficult, as the condition may go unnoticed until it has already caused serious damage to the heart. The appearance of cardiac symptoms and signs in patients who have previously been diagnosed with extra-cardiac sarcoidosis should trigger suspicion of cardiac sarcoidosis.

#### 3.3.2. Biomarkers

To date, multiple circulating and imaging biomarkers have been investigated to allow an early diagnosis of cardiac sarcoidosis (CS), risk stratification of affected patients, monitor disease characteristics during management and follow-up phases, and distinguish between different phenotypes. Despite this, none of the currently available biomarkers meet the criteria for an ideal biomarker. Furthermore, for those that have been most extensively studied and used in clinical practice, their exact roles remain unclear [3,59].

##### Circulating Biomarkers

The main circulating biomarker extensively investigated in systemic sarcoidosis is angiotensin-converting enzyme (ACE); however, limited evidence is available regarding the role of this biomarker in CS [62,63]. Despite the combined use of N-terminal pro-b-type natriuretic peptide (NT-proBNP), serum levels of ACE do not appear to be correlated with typical clinical features of CS, such as arrhythmias [64]. Nevertheless, Komoriyama et al. demonstrated that both high levels of serum ACE and impaired LVEF were associated with a positive EMB, indicating a promising role for serum ACE in the diagnosis of CS [63,65].

High-sensitivity cardiac troponins (hs-cTnT/I) have been described to be frequently elevated in cases of CS upon initial presentation. However, the specificity of these biomarkers for diagnostic purposes has yet to be sufficiently established. Nevertheless, intriguing data are available regarding the potential role of troponins in the management of CS, such as their ability to serve as markers of favorable response to steroid treatment, evidenced by a reduction in troponin levels in responsive patients [65,66].

Another investigated biomarker with a putative role in the diagnosis and risk prognosis of CS is the serum soluble interleukin-2 receptor (sIL-2R). This well-known marker is associated with T lymphocyte activity, but data on its serum levels are limited. However, a study conducted by Kobayashi et al. revealed that high levels of sIL-2R were detectable in CS and were linked to poorer long-term clinical outcomes [67].

Finally, case reports described high levels of lysozyme in CS [68]. Still, conclusive evidence on the role of these biomarkers in diagnosis or risk stratification remains scarce.

##### Imaging Biomarkers

Cardiac involvement in sarcoidosis has different electrophysiological features. Among these, bradycardia, and various degrees of AVB, as well as life-threatening ventricular tachyarrhythmias, are the most commonly reported manifestations [69,70]. However, ECG findings remain variable and mostly nonspecific [3,59].

Overall, echocardiography most frequently identifies unspecific findings, as in other forms of myocarditis. However, certain, more specific, alterations can be suggestive for CS: wall thickness <7 mm (due to parietal fibrosis) or >14 mm (due to inflammation), LV aneurysmal dilatation without signs of cardiac ischemia (frequently localized in inferior and posterior wall), presence of sub-segmental area of hypo/akinesia among segments with normal contractility (typically not respecting the normal coronary distribution) [71,72]. Furthermore, in patients affected by systemic sarcoidosis, the echocardiographic finding of a reduced LVEF (usually less than <40%) can sometimes precede the debut of symptoms in cardiac involvement [71]. Even right ventricular dysfunction can be observed.

CMRI is currently considered an important non-invasive diagnostic tool for CS [32]. The CMRI appearance of CS is highly variable with respect to the stage of the disease and shows varying LGE that can involve all myocardial layers as well as both ventricles, including the right ventricular insertion points [73,74]. Furthermore, it has been shown that CMRI has a complementary value in combination with 18F-FDG PET/CT and adds valuable information concerning risk stratification and prognosis in this challenging patient group [32,34,35]. The presence of myocardial scar has been associated with the development of ventricular arrhythmia and sudden cardiac death [75,76]. In addition, Kouranos et al. (10) showed that the presence of intense LGE on CMRI in patients with CS was an independent predictor of poor outcomes [62].

Nuclear medicine has also proven to be an invaluable diagnostic and management tool for CS. While both 67Ga-citrate scintigraphy and 18F-FDG PET have been utilized for sarcoidosis diagnosis, 18F-FDG PET has demonstrated a higher sensitivity than scintigraphy for the detection of CS [77]. Moreover, 18F-FDG PET is a critical tool for monitoring treatment response by comparing the uptake of 18F-FDG in areas of active inflammation [78,79].

In summary, ECG and echocardiographic abnormalities are variable and typically nonspecific [3,59]. CMRI and FDG PET/CT are considered the most reliable tools for detecting and visualizing CS as well as during follow-up [27,80,81]. Due to the patchy distribution of the disease, EMB has only 20% to 30% sensitivity if not guided by imaging [59]. In patients with extra-cardiac sarcoidosis, a biopsy of the lymph nodes or lungs is preferred due to lower procedural risks and higher sensitivity [59]. However, if an extra-cardiac biopsy is negative, an EMB may be necessary [59].

### 3.4. Giant Cell Myocarditis

#### 3.4.1. Definition, Diagnostic Approach, and Treatment

Giant cell myocarditis (GCM) is a form of rapidly progressing necrotizing myocarditis responsible for 0.5% of myocarditis and 10% of all fulminant myocarditis [82]. This condition is characterized by the presence of multinucleated giant cells in the myocardium and is often associated with a high mortality rate [61,82]. The etiology of GCM remains unclear, and a variety of factors, including viral infections, autoimmune diseases, genetic predisposition, or a combination of these, have been proposed as potential triggers [2]. GCM usually affects middle-aged adults and commonly presents with sudden cardiac symptoms, such as chest pain, shortness of breath, and palpitations [82]. The disease may also present with arrhythmias and HF and may result in sudden cardiac death [24,61].

The diagnosis of GCM is challenging, and the condition is often misdiagnosed; indeed, the clinical presentation of this rare disorder can mimic other inflammatory myocardial disorders. GCM must be suspected in patients with non-ischemic cardiomyopathy with progressive alteration of intraventricular conduction, worsening of cardiac performance, and rapidly progressive HF despite appropriate treatment. A combination of clinical presentation, ECG, and imaging findings can provide important information to support the diagnosis [24]. However, the definitive diagnosis of GCM requires histological confirmation of multinucleated giant cells in the myocardium [3].

Medical therapy, including immunosuppressive and immunomodulatory agents, can lead to clinical remission in up to two-thirds of patients in those not requiring mechanical circulatory support [83,84]. Heart transplantation is an effective therapy in patients with GCM who present with rapidly progressive HF or life-threatening arrhythmias [85].

#### 3.4.2. Biomarkers

Currently, available circulating and imaging biomarkers are not helpful in the diagnosis of GCM. Nevertheless, these biomarkers may play a role in risk stratification and prognosis assessment.

##### Circulating Biomarkers

Among circulating biomarkers related to myocardial injury, troponins are the most investigated in GCM. However, elevated cTnI levels are not indicative of disease duration, and it has been demonstrated that in some cases, even with substantial myocardial tissue necrosis identified by EBM, cTnI levels remain undetectable [86]. As a result, classical biomarkers of myocardial injury are not reliable for GCM diagnosis [86].

Interestingly, cTnT has demonstrated a role in risk stratification, as elevated circulating values of cTnT at myocarditis presentation have been linked to reduced transplant-free survival rates. Ekström et al. observed that elevated circulating values of cTnT (identified cut-off: >85 ng/L) at the presentation of GCM were predictive of death or heart transplantation [84]. Furthermore, troponins, as biomarkers of cardiac injury, and NT-proBNP, as a biomarker of cardiac impairment, were independent predictors of poor outcomes in GCM [87].

It is crucial to note that the clinical and echocardiographic findings of GCM differ depending on whether atrial or ventricular infiltration is prevalent. Atrial fibrillation is widespread among patients with atrial infiltration, while ventricular tachyarrhythmia is the second most common clinical manifestation of GCM [82,87,88]. Data showed high levels of cTnT and NT-proBNP at presentation were associated with ventricular tachyarrhythmias leading to sudden cardiac death during follow-up [89]. Given that GCM carries a high risk of inducing life-threatening ventricular arrhythmia, identifying patients with a high risk of fatal arrhythmia is critical to the clinical management of affected patients.

##### Imaging Biomarkers

Data regarding the specific role of imaging biomarkers in GCM are limited. GCM does not present pathognomonic echocardiographic patterns, and typical findings include LV dysfunction [87]. The degree of LV systolic function impairment can vary significantly, ranging from acute and severe pump function impairment to mild or preserved EF reduction. Other described alterations include wall thickness increase, chamber dimension augmentation with LV dilation and aneurysm formation, as well as thrombosis stratification. Together with LV impairment, the right chambers can also be affected [90]. In some cases, an echocardiogram can be unremarkable [82,87]. The CMRI appearance of GCM is highly similar to CS, showing varying LGE that can involve all myocardial layers as well as both ventricles, including the right ventricular insertion points [91,92]. This makes the differentiation between both entities solely based on CMRI extremely challenging, which underlines the important role of EMB in these cases, as it is the only method that can definitively confirm the diagnosis.

18F-FDG PET/CT might be of help in the challenging differentiation between GCM and CS, as the presence of extra-cardiac sarcoidosis strongly favors the diagnosis of CS. Nonetheless, the role of 18F-FDG PET/CT in patients with suspected GCM is still a matter of debate. A further method enabling molecular imaging of myocardial inflammation is somatostatin receptor PET/CT, which showed promising results in GCM [93].

### 3.5. Eosinophilic Myocarditis

#### 3.5.1. Definition, Diagnostic Approach, and Treatment

Eosinophilic myocarditis (EM) is a rare form of myocardial inflammation characterized by eosinophilic infiltration of the myocardium [4]. The accurate incidence of EM is difficult to define because the diagnosis is often under-recognized and discovered on post-mortem examination [94]. The international registry of histologically proven acute myocarditis with LV systolic dysfunction at presentation reported 29 cases of EM in 220 patients (13%, 19 patients with a fulminant form) [24].

**Figure 5:**
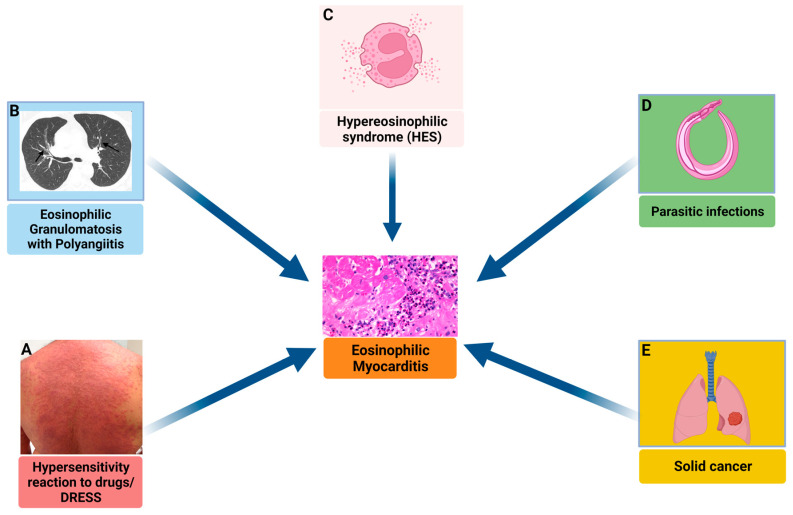
Conditions associated with eosinophilic myocardial injury. Eosinophilic myocarditis exhibits diverse etiologies, ranging from idiopathic to its associated with various systemic disorders. These associated conditions encompass a broad spectrum: (**A**) Hypersensitivity Reactions to Drugs and Drug Reaction with Eosinophilia and Systemic Symptoms (DRESS): These reactions are generally characterized by fever and a diffuse skin rash, often with a delayed onset after drug initiation (typically 2–6 weeks); (**B**) Eosinophilic Granulomatosis with Polyangiitis (EGPA): Often linked with asthma, pulmonary nonfixed infiltrates (indicated by arrows on chest computed tomography in the blue inset), and paranasal sinus abnormalities; (**C**) Hypereosinophilic Syndromes (HES): Distinguished by persistent peripheral eosinophilia (≥1.5 × 10⁹/L for over 6 months), which can manifest as a complex idiopathic form or a myeloproliferative variant; (**D**) Infections caused by parasitic agents; (**E**) rarely, eosinophilic myocarditis may be associated with solid tumors.

The association between eosinophilia and myocardial injury is well established and may present several aetiologies (Figure 5). EM has been reported in association with hypersensitivity reactions [95], immune-mediated disorders, such as eosinophilic granulomatosis with polyangiitis (EGPA) [96], undefined complex hypereosinophilic syndrome (HES) or its myeloproliferative variant [97], infections and cancer [98]. Moreover, in a relatively large number of cases, the underlying cause of EM remains unknown.

Clinical manifestations of EM present a wide spectrum, ranging from mild symptomatology to acute fulminant myocarditis (also called acute necrotizing EM) or chronic restrictive cardiomyopathy (also called Loeffler cardiomyopathy or endo-myocarditis) [98].

The diagnostic suspicion of EM is generally based on clinical presentation, laboratory parameters (elevation of markers of myocardial necrosis and eosinophilia), and imaging, but EMB is the only method that allows a definitive diagnosis [4].

Peripheral eosinophilia is absent in up to 25% of patients with EM, and this feature probably contributes to the underdiagnosis of EM without EMB [98]. Moreover, we know that in EM, eosinophils infiltrate the interstitial compartment and cause necrosis by degranulation of cytotoxic cationic proteins. It is important to remember that achieving a definitive diagnosis of EM is vital in order to begin a specific therapy (i.e., use of cyclophosphamide in EGPA-related EM, imatinib in myeloproliferative variant of PDGFRA-associated HES). This is the case for every subgroup, particularly for HES-associated EM, which is characterized by the highest occurrence of cardiac arrest and in-hospital death [98].

Amongst eosinophilic myocarditis, a specific variant worth discussing is drug reaction with eosinophilia and systemic symptoms (DRESS) syndrome, which is a rare severe T-cell-mediated adverse drug reaction with typical skin rush, hematological abnormality (e.g., eosinophilia or atypical lymphocytosis), and visceral organs involvement [99]. Regarding cardiac complications, myocarditis could develop with delayed onset; notably, alterations of serum biomarkers and abnormal ECG or echocardiographic findings are very nonspecific [100]. This pathological condition is associated with very high mortality, up to 50%, and corticosteroids are the only available treatment [101]. However, its prevalence is very rare, with only 22 cases reported until 2012 [102] and 25 more cases from 2012 to 2018 [101]. The most frequent drugs inducing DRESS myocarditis are minocycline (seven cases), allopurinol (four cases), ampicillin (three cases), dapsone (three cases), and trimethoprim-sulfamethoxazole (TMP-SMX) (two cases) [101].

#### 3.5.2. Biomarkers

##### Circulating Biomarkers

Although peripheral eosinophilia is part of the diagnosis of EM [103], at least 25% of EM cases (and 50% of cases resulting from hypersensitivity mechanisms) can present with normal or only slight elevation of peripheral eosinophilia [104]. In a study including a small number of patients with EM, 75% of patients had an initial eosinophil count of <500/mm^3^, with an increase to ≥500/mm^3^ occurring 7 to 12 days after onset [105]. Therefore, the absence of eosinophilia upon admission does not preclude the diagnosis of EM. These data are in line with findings reported by Brambatti et al. [98], in which among patients without eosinophilia at admission, only 12.8% of patients developed peripheral eosinophilia between the second and sixth day of hospitalization. Elevated CK-MB and troponin levels have been described in patients with EM [103].

Eosinophilic cationic protein (ECP), derived from the degranulation of eosinophils and with a possible mechanistic role, has been described as a biomarker with a high specificity [106]. Elevated ECP serum concentrations are observed in patients with EM, and the levels are associated with the activity of the disease [106]. Intriguingly, ECP may also be useful in the management of EM. Following treatment, a significant decrease in total eosinophil counts and serum levels of ECP was observed in conjunction with a clinical improvement [107]. In addition, after reducing corticosteroid treatment, an increase in the serum ECP levels was identified [107].

##### Imaging Biomarkers

Patients with EM exhibit a wide spectrum of ECG abnormalities, including ST-T changes, AVB, bundle branch blocks, and ventricular arrhythmia. Still, none of these findings is specific to the disease [103].

EM arises from a progressive deterioration of ventricular compliance, ultimately resulting in restrictive cardiomyopathy. The development of eosinophil-mediated damage proceeds through three phases, which are detectable by echocardiography [104,108,109,110] and CMRI [104].

In the initial phase (necrotizing), interstitial myocardial edema causes an increase in wall thickness, with more severe cases featuring LV systolic dysfunction [104,110]. CMRI is particularly helpful in detecting endomyocardial involvement through the use of delayed-enhancement sequences [104]. In the second phase (thrombotic), mural thrombi are detectable along the involved endocardium, causing apical obliteration in both the LV and right ventricle (RV) due to a thrombotic ‘in plus’ image. Thrombosis may also extend to outflow tracts, occasionally reaching the atria. CMRI is more sensitive and specific than echocardiography in detecting these thrombi [104]. In the final phase (fibrotic), there is an advanced reduction in compliance, leading to restrictive diastolic and filling patterns [104]. The progressive involvement of the mitral leaflet, usually the posterior one, with papillary muscle dysfunction results in varying degrees of mitral regurgitation. The prevalence of this finding in subjects investigated is approximately 45% [108,109,111].

CMRI has been shown to play a significant role in community-based screening, such as screening patients with peripheral eosinophilia but no cardiac symptoms for potential cardiac involvement, as well as in phenotyping. In fact, studies have demonstrated that around 20% of patients with eosinophilia and no cardiac symptoms who undergo CMRI have evidence of eosinophilic cardiac disease [112].

Furthermore, CMRI was shown to be able to detect all the different phenotypes of the eosinophilic heart disease spectrum. The most common pattern identified in these cases is the diffuse subendocardial LGE pattern with high signal intensity, which stands in clear contrast to all other forms of myocarditis [112].

These different CMRI findings might also contribute to the risk stratification of patients, with the group with subendocardial LGE patterns displaying the worst outcomes [112].

Finally, both echocardiography and CMRI can be used to monitor the response to immunosuppressive treatment, following the changes over time during appropriate treatment strategies [112,113].

### 3.6. Check Point Inhibitors Myocarditis

#### 3.6.1. Definition, Diagnostic Approach, and Treatment

Immune checkpoint inhibitors (ICIs) are monoclonal antibodies that inhibit the immune checkpoints CTLA-4 (cytotoxic T-lymphocyte antigen-4), PD-1 (programmed death receptor-1), and PD-L1 (programmed death ligand-1), and are increasingly used in cancer therapies [114]. Immune system activation, achieved by blocking immune checkpoints, allows T-cells to target tumors [115]. Tumor response can be strengthened by using a combination therapy [114]. While these therapies have been shown to have an excellent response in both solid and hematological malignancies, ICIs carry a risk of immune-related adverse events [116] and are a known cause of myocarditis and pericarditis [117].

The true incidence of ICI-myocarditis is unknown due to the lack of validated monitoring criteria [118]. The reported prevalence of ICI-myocarditis is 1.14%, increasing to 2.4% in patients undergoing combination ICI therapy with PD-1 and CTLA-4 [115], and increases the risk of other cardiovascular effects [116].

The mechanism for ICI-myocarditis has not yet been fully elucidated; however, it is thought to occur due to molecular mimicry between tumor antigens and antigens found in the myocardium [115]. This molecular mimicry may occur either between an antigen present in either cardiomyocytes and tumor cells or two unique antigens of a similar structure. Alternatively, T-cells may be targeting different antigens by different receptors [118]. EMB reports have demonstrated that ICI-myocarditis is characterized by T-cell infiltration [115,118], consistent with the theory that ICI-myocarditis is mediated by T-cells.

The median time of presentation of ICI-myocarditis is reported as 6 weeks from starting ICI therapy [116]. The clinical presentation of ICI-myocarditis can range from asymptomatic with biomarker changes to acute coronary syndrome-like symptoms to fulminant presentations associated with ventricular arrhythmias, multiorgan failure, and cardiac arrest [118]. ECG abnormalities appeared to be more common in ICI-myocarditis (89%) [115], compared to 74% in a CMRI-proven acute myocarditis cohort [28].

ICI-myocarditis can be diagnosed using the European Society of Cardiology (ESC) diagnostic framework [4] and the “Updated Lake Louise Criteria” for CMRI [26].

Initial management of ICI-myocarditis includes supportive management, stopping ICI treatment, and initiation of corticosteroids [114,116]. Retrospective data show that patients will benefit more from early initiation of high-dose corticosteroids [119], in particular intravenous methylprednisolone [114]. Case reports of successful treatment of ICI-myocarditis included the use of Mycophenolate Infliximab, antithymocyte globulin, intravenous immunoglobulin [115], Tacrolimus and extracorporeal membrane oxygenation (ECMO) support [120]. The prognosis of ICI-myocarditis is reported to be poor, with mortality ranging from 30–50% [119].

#### 3.6.2. Biomarkers

To date, no specific circulating or imaging biomarkers are available with regard to ICI-myocarditis.

##### Circulating Biomarkers

As in other ICM, elevated biomarkers of myocardial injury, such as troponins, and cardiac impairment, such as natriuretic peptides, have been observed in patients with ICI-myocarditis, but their specific roles are unclear. In particular, elevated levels of natriuretic peptides have been found in ICI-myocarditis, but these biomarkers may also be elevated in cancer patients without cardiac impairment, which limits their diagnostic utility [121].

Furthermore, CK levels have been linked to myositis in ICI-myocarditis [121]. As such, the presence of increased CK levels in a patient with ICI-myocarditis raises suspicion for concomitant myositis. However, the specificity of CK levels is relatively low, and a normal CK value cannot be used to rule out myocarditis [121].

##### Imaging Biomarkers

With regard to ECG findings, about 90% of patients with ICI-myocarditis displayed abnormalities. Typically, ECG abnormalities represent the first alteration suggestive of myocardial inflammation, although their specificity is limited, precluding a definitive diagnosis. The most common ECG abnormalities in patients undergoing ICI therapy include atrial fibrillation, atrioventricular conduction abnormalities, and ventricular fibrillation [118,122,123].

On the other hand, echocardiography reveals that patients with ICI-myocarditis rarely experience a reduction in LVEF, with most patients exhibiting preserved EF. Nonetheless, a speckle-tracking echocardiography-derived global longitudinal strain can reveal myocardial dysfunction in these patients [121]. The CMRI appearance of ICI-myocarditis is usually less pronounced than in other more common forms of myocarditis, and the CMRI study can even be non-diagnostic. LGE may not appear immediately on CMRI (21.6% LGE detected in CMRI within 4 days of presentation versus 72.0% in CMRI after 4 or more days post presentation). Consequently, a non-diagnostic CMRI taken within 4 days of presentation should not automatically exclude myocarditis [119]. Extra care should be taken in patients with concomitant myositis, where T2-weighted imaging may give a false negative result, as skeletal muscle is used as a reference to determine the presence of edema [124].

## 4. Future Perspectives and Research

Other biomarkers have been investigated in the setting of myocarditis and ICM. However, they have not yet entered into clinical practice, and/or their current role is limited to the research field or only in referral centers. The following section provides a brief outline of the most promising biomarkers to date.

### 4.1. Liquid Biopsy

#### 4.1.1. Micro-RNA

Micro-RNA (mi-RNA), short non-coding RNAs transcribed from DNA and processed into mi-RNA, have a critical role in regulating gene expression through messenger RNA (mRNA) and have been extensively investigated in the cardiovascular field [125,126].

A recent systematic review based on available literature screened a total of 187 different mi-RNAs, with eight being assessed as “very high” and 23 as “high” utility in myocarditis and ICM [127]. Of these, three mi-RNAs (miR-Chr8:96, miR-155, and miR-206) have demonstrated the highest potential as biomarkers of myocardial inflammatory state. Serum levels of these mi-RNAs have been associated with the diagnosis of ICM and exhibit high specificity [127]. Intriguingly, by combining several mi-RNA levels, the accuracy of liquid biopsy diagnostics can be significantly improved [128].

#### 4.1.2. Circulating Cell-Free DNA

Circulating cell-free DNA (cfDNA), mostly studied in the field of oncology, is genomic DNA released as a consequence of cell death mechanisms, such as apoptosis, necrosis, and autophagy [129]. To date, cfDNA fragments have demonstrated a crucial role in the early detection, treatment monitoring, and prognosis/risk stratification of patients affected by cancer [129,130,131]. In addition, an association has recently been shown between cfDNA and several cardiovascular risk factors, [132] cardiovascular diseases (i.e., acute myocardial infarction, atrial fibrillation, and HF) [133,134], as well as early diagnosis of heart transplant rejection [135,136,137], potentially replacing the use of EBM. In the field of ICM, it has been widely speculated that cfDNA may provide a valuable tool in the detection, clinical management, and risk stratification of ICM [127]. However, no data are currently available, indicating the need for further development in this future research field.

### 4.2. Soluble ST2 Receptors

Soluble IL-1 receptor-like 1, also known as soluble suppression of tumorigenesis-2 (s-ST2), belongs to the Toll-like/interleukin-1 receptor superfamily, binding to IL33, and has been shown to have antihypertrophic and antifibrotic effects on cardiomyocytes [138].

In the context of ICM, a possible role of s-ST2 in risk stratification has been demonstrated. Specifically, it has been shown, with age and sex difference (in young, ≤50 years, and male subjects), that elevated levels are associated with an increased risk of more severe HF, as assessed by the New York Heart Association (NYHA) class [138]. Recent studies have also shown the superiority of s-ST2 levels, when compared to natriuretic peptides or troponins, in association with the degree of LV impairment and functional NYHA class at mid-term follow-up [139].

### 4.3. Galectin-3

Galectin-3, a β-galactoside-binding lectin known for its proinflammatory effects, has emerged as a promising biomarker in HF and has recently been investigated in ICM [140]. In patients with ICM, myocardial Galectin-3 expression was significantly associated with the inflammatory cell count on EMB, with an inverse relationship with cardiac fibrosis. Of note, circulating Galectin-3 levels did not correlate with myocardial Galectin-3 levels or LV fibrosis. Hence, a positive correlation between circulating Galectin-3 levels and inflammatory cell count on EMB was observed [140].

### 4.4. Molecular Inflammation Imaging Using PET

Several novel PET tracers are currently under investigation to enable a better diagnosis of myocarditis with a special focus on cardiac sarcoidosis [141]. Promising results have, amongst others, been shown for somatostatin receptor and folate receptor β-targeted PET/CT [142,143].

### 4.5. Cardiac Autoantibodies (aabs)

Despite acute myocarditis being resolved in about 50% of cases in the first 2–4 weeks and about 70% of patients recovering myocardial function in the first year, 25–30% of patients display a progression from myocarditis to dilated cardiomyopathy [4]. Specifically, patients with persistent (chronic) inflammation [144] are at higher risk of displaying a poor outcome. Notably, it has been shown that several of these patients developed pathogenic cardiac autoantibodies [4], which are directed against myocardial structural, sarcoplasmic, or sarcolemmal proteins. These serum cardiac autoantibodies are found in myocarditis and DCM with different frequencies, and there is a different disease specificity for such antibodies [4]. With regard to their clinical use, it is of interest that when detected, together with the lack of viral genome on EMB, an immune-mediated DCM or inflammatory myocarditis can be suspected.

In addition, they also have a role in the choice of treatment management; indeed, in patients with detectable cardiac autoantibodies, in the absence of active infection of the myocardium, immunosuppression and/or immunomodulation may be considered, with some beneficial effects [4]. Some of these biomarkers are associated with a poor prognosis.

Finally, they can also be used as screening biomarkers to identify patients’ relatives at risk of developing a DCM.

In brief, limiting to cardiac autoantibodies with evidence in both myocarditis and dilated cardiomyopathies, it is possible to identify

- Muscle-specific anti-sarcolemmal (ASA; i.e., AFA, anti-fibrillary, IFA, anti-interfibrillary aabs, and AMLA, anti-myolemmal aabs), index of myocytolysis and with a prevalence ranging 28–59% in myocarditis and 9–41% in DCM;
- Cardiac-specific (AHA, organ-specific and partially organ-specific anti-heart aabs [4]; AIDA, anti-intercalated disks-aabs, and anti-alpha-myosin heavy chain, MHC) [145,146], early predictors of disease, and able to predict DMC development in relatives, with a prevalence ranging 17–56% in myocarditis and 16–30% in DCM [145,146];
- Anti-beta 1- adrenergic receptors (33–96% and 27–95% respectively in myocarditis and DCM), associated with a negative prognosis and in vitro pro-apoptotic effects [4];
- Anti-muscarin acetylcholine receptor-2 (11% and 30–83% respectively), with negative inotropic, muscarin effects and associated with atrial arrhythmia [4];
- Anti-lamin (73% and 78%) [147];
- Anti-ANT, adenine nucleotide translocator, with negative inotropic effects (91% and 57%) [148];
- Anti-M7, against mitochondria (13% and 31%) [149];
- Anti-BCKD-E2, branched chain alpha-ketoacid dehydrogenase dihydrolipoyl transacylase (100% and 60%) [150].

However, they are not routinely available in clinical practice, are limited to tertiary referral centers, and their use still needs to be expanded [4].

## 5. Gaps in Evidence and Conclusions

To date, the role of biomarkers, both circulating and imaging, in myocarditis and ICM remains controversial (Table 1). Excluding endomyocardial biopsy, currently available biomarkers for acute myocarditis and ICM have limited sensitivity and specificity for the diagnosis. Furthermore, it remains a challenge to discriminate between different phases, such as acute, subacute, chronic, or sequelae, as well as between the different possible aetiologies of myocarditis/ICM. In addition, it is generally not possible to identify the specific role of each biomarker at different stages of the disease continuum, including community-based screening, diagnosis, risk stratification/prognosis, phenotyping, management/monitoring, and treatment. Moreover, there are limited studies with longitudinal data with biomarkers measured at different time points of the disease. Finally, most of the available biomarkers are not pathognomonic of specific cardiomyopathy and are rather altered to varying degrees in all ICMs. Therefore, future research should aim to fill the aforementioned gaps in evidence, with a particular focus on providing clinicians with sensitive and specific biomarkers/combinations of biomarkers able to strongly support the diagnosis of myocarditis and enable a reliable risk stratification. These biomarkers could potentially guide the management of patients affected by acute myocarditis and chronic ICM.

## Figures and Tables

**Table 1:** Role of circulating and imaging biomarkers in ‘biomarkers continuum’ in Acute Myocarditis and Chronic Inflammatory Cardiomyopathy.

	Community Screening	Diagnosis	Phenotyping	Risk Stratification	Management	Treatment
**Infectious Myocarditis** [2,3,4,5,6,8,13,14,17,18,22,23,24,25,37,38,39,40,41,42,43,44,45,46,47]	CK CK-MB Troponins	Blood cell count CRP Erythrocyte sedimentation rate Troponins Virus serology	IgM IgG	Troponins NP CA-125 * Uric acid * CRP * IL-8 * IL-1b * IL-12 * Mir 223-5p *	Troponins NP	CRP
		TTE CMRI		TTE		
**COVID-19 and Post-vaccination Associated Myocarditis** [26,48,49,50,51,52,53,54,55]		BNP CRP Troponins				
		ECG CMRI				
**Sarcoidotic Myocarditis** [3,10,23,31,51,53,56,57,58,59,60,61,62,63,64,65,66,67,68,69,70,71,72,73,74,75,76]	ACE	ACE Lysozyme NT-pro-BNP Troponins		Lysozyme sIL-2R	sIL-2R Troponins	Troponins
		ECG TTE CMRI ^67^Ga-citrate scintigraphy ^18^F-FDG PET		CMRI ^18^F-FDG PET	^67^Ga-citrate scintigraphy ^18^F-FDG PET	^18^F-FDG PET
**Giant Cell Myocarditis** [58,72,73,74,75,76,77,78,79,80,81,82,83,84,85,86,87,88,89,90]		Troponins		hs-cTnT NT-pro-BNP	hs-cTnT NT-pro-BNP	hs-cTnT
		TTE CMRI ^18^F-FDG PET ^¶^				
**Eosinophilic Myocarditis** [91,92,93,94,95,96,97,98,99,100,101,102,103,104,105,106]	Peripheral eosinophilia	CK-MB ECP Peripheral eosinophilia Troponins	CMRI		ECP	ECP Peripheral eosinophilia
	CMRI	ECG TTE CMRI			TTE CMRI	TTE CMRI
**Check Point Inhibitors** [107,108,109,110,111,112,113,114,115,116,117]		CK Troponins NP			CMRI	
		ECG CMRI TTE				
**Future Perspectives and Research** [118,119,120,121,122,123,124,125,126,127,128,129,130,131,132,133,134,135,136,137,138,139,140,141,142,143]		miR-Chr8:96 miR-155 miR-206 CMRI Novel PET tracers for inflammation imaging, e.g., SSTR PET/CT		cfDNA s-ST2 CMRI Novel PET tracers for inflammation imaging, e.g., SSTR PET/CT	cfDNA Galectin-3 CMRI Novel PET tracers for inflammation imaging, e.g., SSTR PET/CT	cfDNA

Abbreviations: ACE, angiotensin-converting enzymes; AF, atrial fibrillation; AV, atrio-ventricular; BNP, brain peptide natriuretic; cfDNA, Circulating cell-free DNA; CRP, C-reactive protein; CT, computed tomography; ECP, eosinophilic cationic protein; ECG, electrocardiogram; hs-cTNT/I, High sensibility cardiac troponins; CMRI, Cardiac magnetic resonance imaging; GLS, global longitudinal strain; LV, left ventricular; LVEF, left ventricular ejection fraction; mi-RNA, micro-RNA; NP, natriuretic peptides; NT-pro-BNP, N-terminal pro b-type natriuretic peptide; PET, Positron emission tomography; sIL-2R, serum soluble interleukin-2 receptor; s-ST2, soluble ST2 Receptor; SSTR, somatostatin receptor; TTE, transthoracic echocardiography; VF, ventricular fibrillation; 18F-FDG, 18F-fluorodeoxyglucose. * Biomarkers used in Chagas’ disease; ^¶^ To differentiate giant cell myocarditis from cardiac sarcoidosis.
